# Heterologous expression of the *N*-acetylglucosaminyltransferase I dictates a reinvestigation of the *N*-glycosylation pathway in *Chlamydomonas reinhardtii*

**DOI:** 10.1038/s41598-017-10698-z

**Published:** 2017-08-31

**Authors:** Gaëtan Vanier, Pierre-Louis Lucas, Corinne Loutelier-Bourhis, Jessica Vanier, Carole Plasson, Marie-Laure Walet-Balieu, Philippe Chan Tchi-Song, Isabelle Remy-Jouet, Vincent Richard, Sophie Bernard, Azeddine Driouich, Carlos Afonso, Patrice Lerouge, Elodie Mathieu-Rivet, Muriel Bardor

**Affiliations:** 1 Normandie Univ, UNIROUEN, Laboratoire Glycobiologie et Matrice Extracellulaire végétale, EA 4358, 76000 Rouen, France; 2 Normandie Univ, UNIROUEN, COBRA, UMR 6014 et FR 3038, Université de Rouen, INSA de Rouen, CNRS, 76000 Rouen, France; 3 Normandie Univ, UNIROUEN, Plate-Forme de Protéomique PISSARO, 76000 Rouen, France; 4 Normandie Univ, UNIROUEN, Institut de Recherche et d’Innovation Biomédicale (IRIB), 76000 Rouen, France; 5 Normandie Univ, UNIROUEN, Inserm UMR 1096, Plateforme BOSS, 76000 Rouen, France; 6 Normandie Univ, UNIROUEN, Plate-forme, PRIMACEN, Cell Imaging Platform of Normandy, 76000 Rouen, France; 7 Institut Universitaire de France (I.U.F.) 1, rue Descartes, 75231 Paris, Cedex 05, France; 8 UMR FARE 614, Fractionnement des AgroRessources et Environnement, Chaire AFERE, Université de Reims-Champagne-Ardenne, INRA, 51686 Reims Cedex, France

## Abstract

Eukaryotic *N*-glycosylation pathways are dependent of *N*-acetylglucosaminyltransferase I (GnTI), a key glycosyltransferase opening the door to the formation of complex-type *N*-glycans by transferring a *N*-acetylglucosamine residue onto the Man_5_GlcNAc_2_ intermediate. In contrast, glycans *N-*linked to *Chlamydomonas reinhardtii* proteins arise from a GnTI-independent Golgi processing of oligomannosides giving rise to Man_5_GlcNAc_2_ substituted eventually with one or two xylose(s). Here, complementation of *C. reinhardtii* with heterologous GnTI was investigated by expression of GnTI cDNAs originated from Arabidopsis and the diatom *Phaeodactylum tricornutum*. No modification of the *N-*glycans was observed in the GnTI transformed cells. Consequently, the structure of the Man_5_GlcNAc_2_ synthesized by *C. reinhardtii* was reinvestigated. Mass spectrometry analyses combined with enzyme sequencing showed that *C. reinhardtii* proteins carry linear Man_5_GlcNAc_2_ instead of the branched structure usually found in eukaryotes. Moreover, characterization of the lipid-linked oligosaccharide precursor demonstrated that *C. reinhardtii* exhibit a Glc_3_Man_5_GlcNAc_2_ dolichol pyrophosphate precursor. We propose that this precursor is then trimmed into a linear Man_5_GlcNAc_2_ that is not substrate for GnTI. Furthermore, cells expressing GnTI exhibited an altered phenotype with large vacuoles, increase of ROS production and accumulation of starch granules, suggesting the activation of stress responses likely due to the perturbation of the Golgi apparatus.

## Introduction

*N*-linked glycosylation is an extensive eukaryotic post-translational modification of secreted proteins consisting of the covalent attachment of an oligosaccharide onto asparagine residues belonging to the consensus sequence Asn-X-Ser/Thr/Cys, where X represents any amino acid except proline^1–4^. The process starts in the endoplasmic reticulum (ER) with the biosynthesis of a lipid-linked oligosaccharide (LLO) precursor which involves the action of a conserved set of enzymes named Asparagine-Linked Glycosylation (ALG). First steps of the LLO synthesis occur on the cytosolic face of the ER membrane where *N*-acetylglucosamine (GlcNAc) and mannose (Man) residues are added step by step onto a membrane-anchor dolichol pyrophosphate (Dol-PP) to form a Man_5_GlcNAc_2_-PP-Dol. Then, the Man_5_GlcNAc_2_-PP-Dol is flipped into the lumen of the ER^5, 6^ where its extension occurs by the addition of several Man and glucose (Glc) residues into a complete oligosaccharide precursor Glc_3_Man_9_GlcNAc_2_-PP-Dol^7^. This LLO is thereafter transferred by the oligosaccharyltransferase (OST) complex onto the asparagine of the *N*-glycosylation consensus sequence of the nascent polypeptides^8^. The *N*-glycan is then trimmed by the action of α-glucosidases I and II, and eventually an ER-mannosidase into Man_8-9_GlcNAc_2_ oligomannoside. During these ER events, interactions between the glycoprotein and ER-resident chaperones ensure the glycoprotein folding^9, 10^. Correctly folded glycoproteins then enter the Golgi apparatus where their *N*-glycans are processed through the action of an α-mannosidase I into Man_5_GlcNAc_2_. Subsequently, *N-*acetylglucosaminyltransferase I (GnTI), α-mannosidase II and finally *N*-acetylglucosaminyltransferase II (GnTII) give rise to the canonical GlcNAc_2_Man_3_GlcNAc_2_ core common to mammals, insects and land plants^11, 12^. This core undergoes further maturation into organism-specific complex-type *N*-glycans that are involved in several physiological functions like cell-cell interaction, intracellular communication and signaling^13, 14^.

The structures of *N*-linked glycans of the green microalgae *C. reinhardtii* were recently characterized^15^. Predominant *N*-glycans carried by *C. reinhardtii* endogenous proteins are oligomannosides ranging from Man_2_GlcNAc_2_ to Man_5_GlcNAc_2_. In addition, mature *N-*glycans were identified as oligomannosides Man_4_GlcNAc_2_ and Man_5_GlcNAc_2_ presenting methylation on mannose residues and substituted by one or two xylose residues^15^. The absence of terminal GlcNAc residues on these *N*-linked glycans suggested that mature *N*-glycans in *C. reinhardtii* are derived from GnTI-independent Golgi events^11, 15^. *In silico* analysis of the *C. reinhardtii* genome allowed the identification of numerous candidate genes encoding enzymes of the protein *N*-glycosylation pathway, such as the ALG, OST subunits, glycosidases and glycosyltransferases involved either in the ER or Golgi *N*-glycan biosynthesis steps^11, 15^. However, in agreement with biochemical analyses, no GnTI candidate is predicted in *C. reinhardtii* genome suggesting that the *N*-glycosylation pathway in this organism is different from that found in land plants, insects, mammals^12, 16^ and diatoms for which a functional GnTI has been recently characterized^17^.

Considering the difference in the *N*-glycosylation pathways between *C. reinhardtii* and land plants, as well as between *C. reinhardtii* and diatoms, we have undertaken the expression of either a diatom or a plant GnTI in *C. reinhardtii* to investigate the effects of such a complementation on the physiology and *N*-glycan pathway in *C. reinhardtii*. Our findings demonstrate that the expression of GnTI induces stress responses in *C. reinhardtii* and reveal a truncated ER *N*-glycosylation pathway in *C. reinhardtii*.

## Results

**Figure 1:**
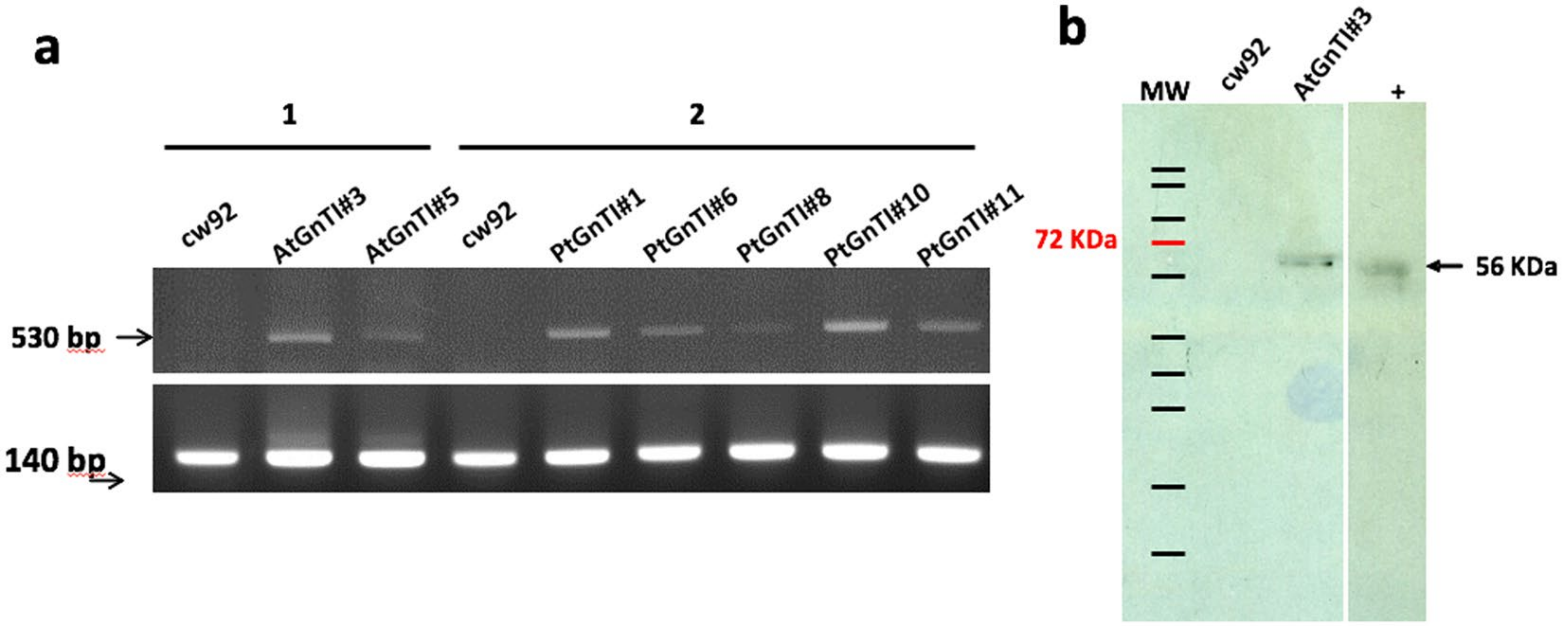
(**a**) RT-PCR analysis of *GnTI* transcription level in cw92 cells, cell lines transformed with *AtGnTI-V5* (lines AtGnTI#3 and AtGnTI#5) or *PtGnTI*-*V5* (lines PtGnTI#1, PtGnTI#6, PtGnTI#8, PtGnTI#10 and PtGnTI#11) using *AtGnTI* (1) or *PtGnTI* (2) specific primers. Actin (lower panel) was used as an RT-PCR control. (**b**) Immunodetection of recombinant GnTI in the microsomal fraction isolated from cw92 cells and AtGnTI#3 lines respectively. The immunodetection was performed using an anti-V5 antibody as a primary antibody. A protein extract from CHO cells expressing *PtGnTI-V5* (+) was used as a positive control^17^. Full images of the agarose gel and the Western blot are presented in Figs S1 and S2.

The commonly used cw92 laboratory strain was transformed with the codon optimized sequences encoding for the catalytically active GnTI from Arabidopsis (At*GnTI*) or from the diatom *Phaeodactylum tricornutum* (Pt*GnTI*). Both have been shown to be able to process *N*-linked glycans. At*GnTI* (At4g38240) encodes for a Golgi enzyme that is responsible for the transfer of a terminal GlcNAc residue onto Man_5_GlcNAc_2_ *N*-glycan^18, 19^. PtGnTI has been shown to restore *in vivo* the biosynthesis of complex-type *N*-glycans in CHO Lec1 mutant that lacks endogenous GnTI activity^17^. These sequences fused to a tag sequence encoding for a V5-epitope were used for nuclear expression in *C. reinhardtii* ^20–22^. RT-PCR analyses show that both plant and diatom *GnTI* were expressed in *C. reinhardtii* transformed lines (Fig. 1 and Fig. S1). Further experiments were focused on two At*GnTI* and four Pt*GnTI* expressing lines, all of which exhibit the highest transcription levels (Fig. 1a). Furthermore, using anti-V5 epitope antibodies, a signal around 56 kDa was immunodetected on a western blot in the microsomal fraction isolated from the transgenic lines but not in that of the non-transformed cells (Fig. 1b and Fig. S2).

**Figure 2:**
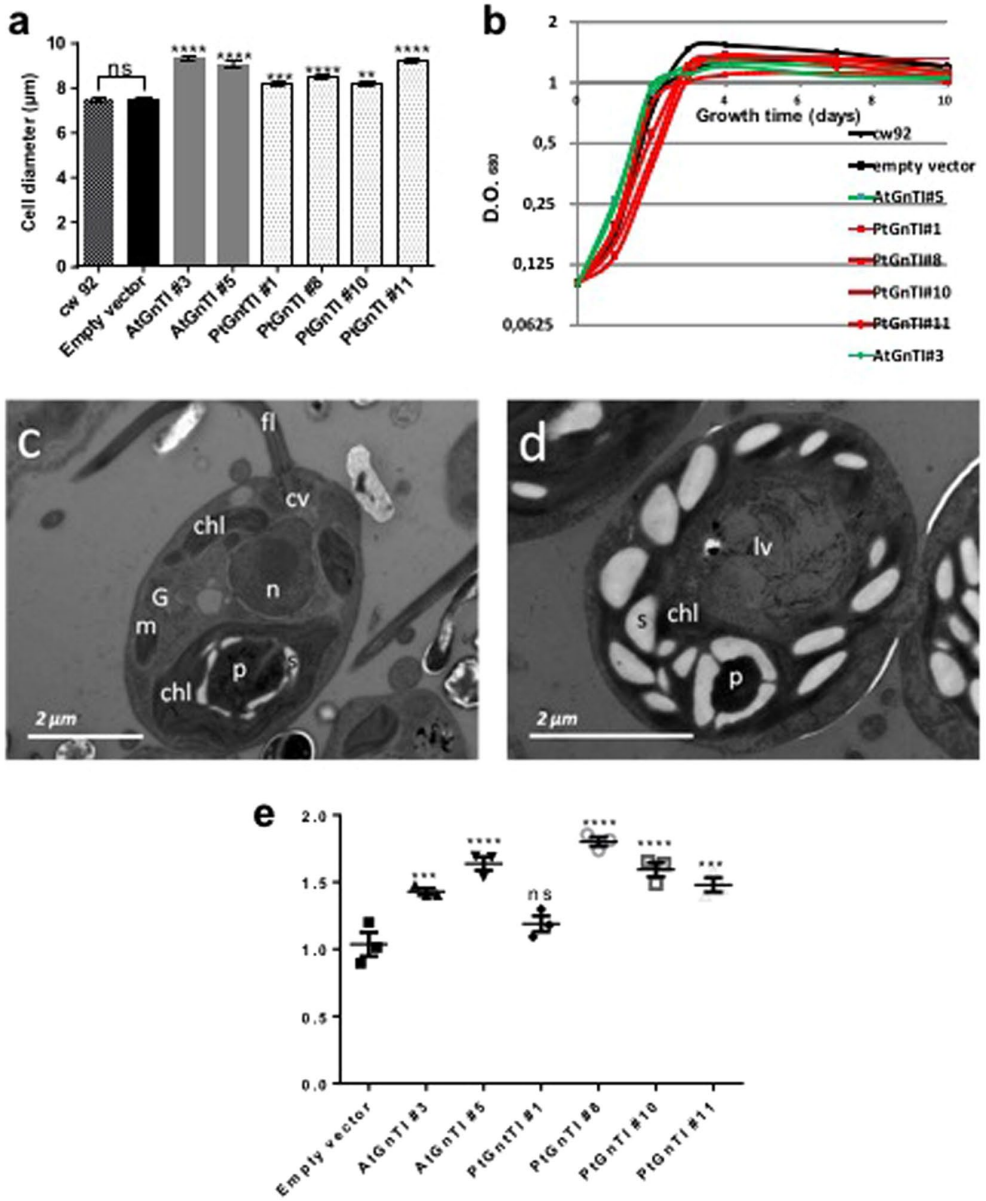
(**a**) Measurement of the cell diameters of *C. reinhardtii* cells expressing *AtGnTI* or *PtGnTI* as compared to the cw92 cells and cells transformed with an empty vector (Kruskal-Wallis test with n > 200 and p-value fixed at 0.05; stars indicate the significant level of the test). (**b**) Growth rate of cw92 and transformed cell lines grown in TAP medium. (**c,d**) Ultrastructure of cw92 cells (**c**) and transformed cell line AtGnTI#3 (**d**) by Transmission Electron Microscopy (TEM). chl: chloroplast, fl: flagella, G: Golgi apparatus, m: mitochondrion, n: nucleus, P: pyrenoid, s: starch granules, cv: contractile vacuoles, lv: large vesicles. (**e**) ROS levels in transformed cell lines determined through the oxidation measurement of CMH spin probe by electron paramagnetic resonance spectroscopy. The ROS level (arbitrary units/12 × 10^4^ cells hour^−1^) in each transformed cell line was normalized against ROS level measured in cw92 cells. After normalization, a statistical test was performed between GnTI expressing cell lines and cells transformed with the empty vector using Ordinary One-Way ANOVA with n = 3 and p-value fixed at 0.05.

The phenotypic analysis of *C. reinhardtii* transformed cell lines revealed that the expression of At*GnTI* or Pt*GnTI* was correlated to a modification of the cell size. Indeed, measurements of longitudinal cell diameters showed that cells expressing At*GnTI* or Pt*GnTI* were statistically enlarged as compared to the cw92 cells and cells transformed with the empty vector (Fig. 2a). Despite the size difference, the growth rates of transformed cells were similar to those of cw92 cells indicating that the swelling observed in transformed lines did not alter cell growth (Fig. 2b).

To examine whether the ultrastructure of swollen cells is altered, cells were high pressure frozen and analyzed by thin-section electron microscopy. To further characterize the cell swelling, the ultrastructure of the transformed lines was analyzed by Transmission Electron Microscopy (TEM). As illustrated in Fig. 2c,d, GnTI expressing cells exhibited a large number of starch granules. In addition, large vesicles appeared in the GnT I expressing lines as compared to the cw92 and cells transformed with the empty vector. In order to check if the increase in size and the starch accumulation were associated to a stress phenotype in transformed cells, we measured the production of reactive oxygen species (ROS) in the different lines using electron paramagnetic resonance (EPR) spectroscopy. Figure 2e shows that ROS levels were significantly higher in the cells expressing At*GnTI* or Pt*GnTI* than in the cw92 cells, except for PtGnTI#1 line, in which ROS content was not significantly increased.

Protein *N*-glycosylation in cw92 and transformed cells were then investigated by mass spectrometry profiling. *N*-linked glycans were released from total protein extracts using PNGase F, labeled with 2-aminobenzamide (2AB) and analyzed by liquid chromatography coupled to electrospray ionization mass spectrometer (LC-ESI-MS). Although the expression of GnTI induced altered phenotypes in *C. reinhardtii*, the comparison of the *N*-glycan structures between cw92 cells and the transformed lines did not reveal any modification of the *N*-glycan profiles (Fig. S3). A specific search for mono- and discharged ions corresponding to *N-*glycans exhibiting extra terminal GlcNAc residue was unsuccessful. This suggested that the expression of At*GnTI* or Pt*GnTI* did not affect, in a detectable manner, the *N*-glycosylation of endogenous proteins in *C reinhardtii*.

**Figure 3:**
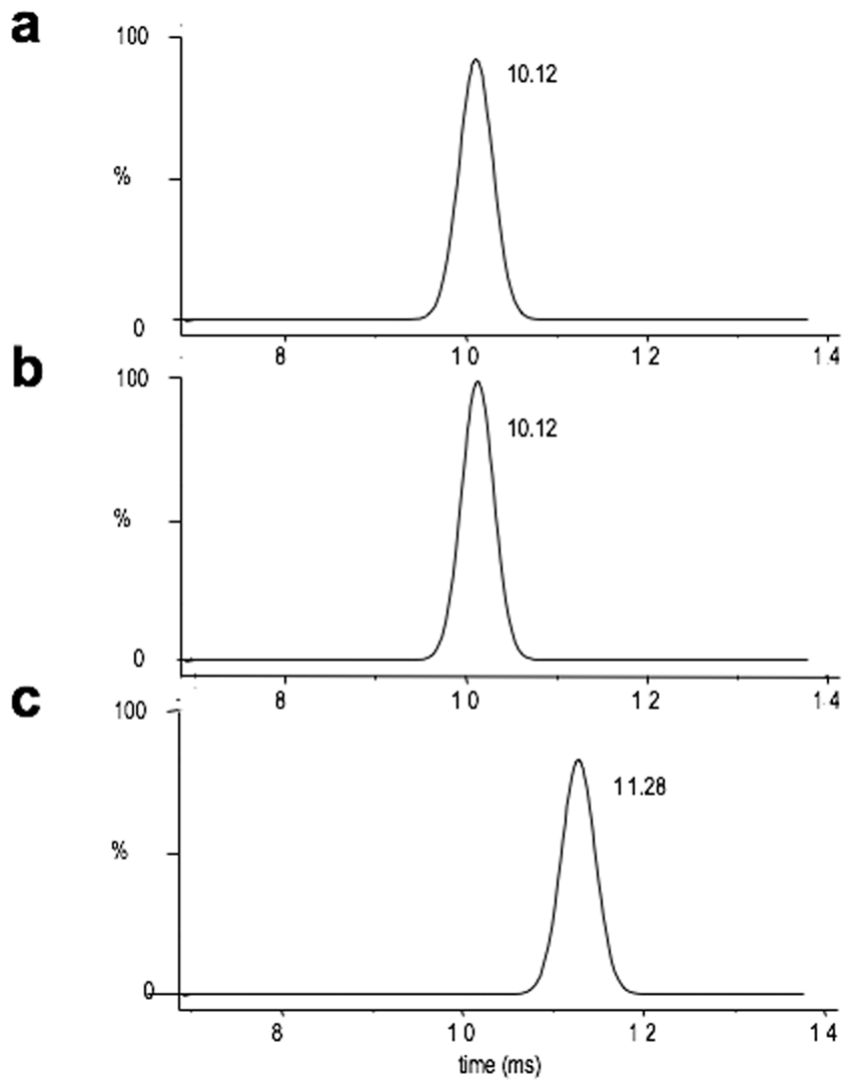
(**a**–**c**) Ion mobility spectra of Man_5_GlcNAc_2_-2AB derivatives prepared from two independent preparations of *C. reinhardtii* proteins (**a**,**b**) and bovine ribonuclease B (**c**) (M + Na^+^ adducts).

Different scenarios may explain the inability of exogenous GnTI to affect the protein *N*-glycan profiles in *C. reinhardtii*. This may result from a very weak protein expression of active GnTI in the Golgi apparatus or its mislocalization in this organelle. Absence of the appropriate substrates in the Golgi apparatus could also explain the GnTI inactivity. This concerns the nucleotide-sugar (UDP-GlcNAc) or the oligomannoside Man_5_GlcNAc_2_ *N*-linked to secreted proteins. Man_5_GlcNAc_2_ oligomannoside was previously identified in *C. reinhardtii* cw92 proteins and was assigned to a branched Man_5_GlcNAc_2_ by analogy with data published regarding the Golgi *N*-glycan processing in eukaryotes^15^. The non-effect of GnTI on the *N*-glycan profiles from *C. reinhardtii* raised questions regarding the structure of this Man_5_GlcNAc_2_, which therefore needs to be reinvestigated. Here, we made use of Ion Mobility Spectrometry–Mass Spectrometry (IMS-MS), a reliable analytical method which allows the separation of isomers including oligosaccharide isomers^23–25^. In this analysis, the ion mobility of Man_5_GlcNAc_2_ coupled to 2AB oligosaccharide (sodium adduct) prepared from *C. reinhardtii* proteins was compared to the ion mobility of branched sodiated 2AB labeled Man_5_GlcNAc_2_ obtained from the bovine ribonuclease B. As illustrated in Fig. 3, Man_5_GlcNAc_2_-2AB from *C. reinhardtii* and from bovine ribonuclease B exhibited different ion mobilities (drift time of 10.12 ms and 11.28 ms, respectively) which suggested that they possess distinct structures. This data indicated that Man_5_GlcNAc_2_ in *C. reinhardtii* is different from the branched Man_5_GlcNAc_2_ that results from the trimming of oligomannosides Man_8-9_GlcNAc_2_ in the ER and the Golgi apparatus in plants and mammals.

**Figure 4:**
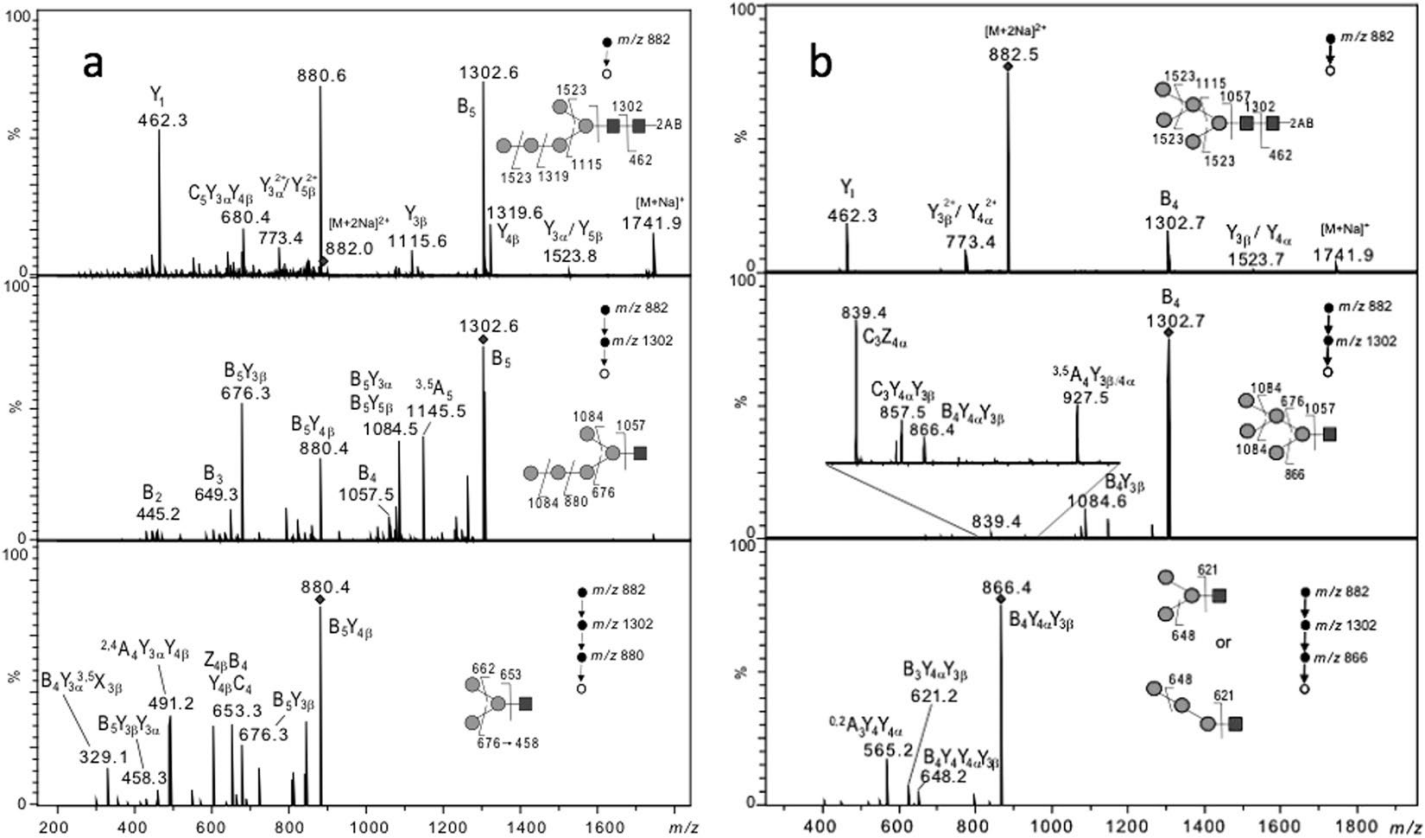
ESI-MS^n^ spectra with n = 2 (upper panel), n = 3 (middle panel) and n = 4 (lower panel) of permethylated Man_5_GlcNAc_2_-2AB derivative (*m/z* 882 corresponding to [M + 2Na]^2+^ precursor ion) isolated from PtGnTI *C. reinhardtii* proteins (**a**) and from Ribonuclease B (**b**) On each panel, the precursor ion selected for the fragmentation analysis is shown with a diamond and its fragmentation pattern is proposed according to one of the possible structures. 2AB: 2-aminobenzamide; Black square: GlcNAc; grey circle: Man.

**Figure 5:**
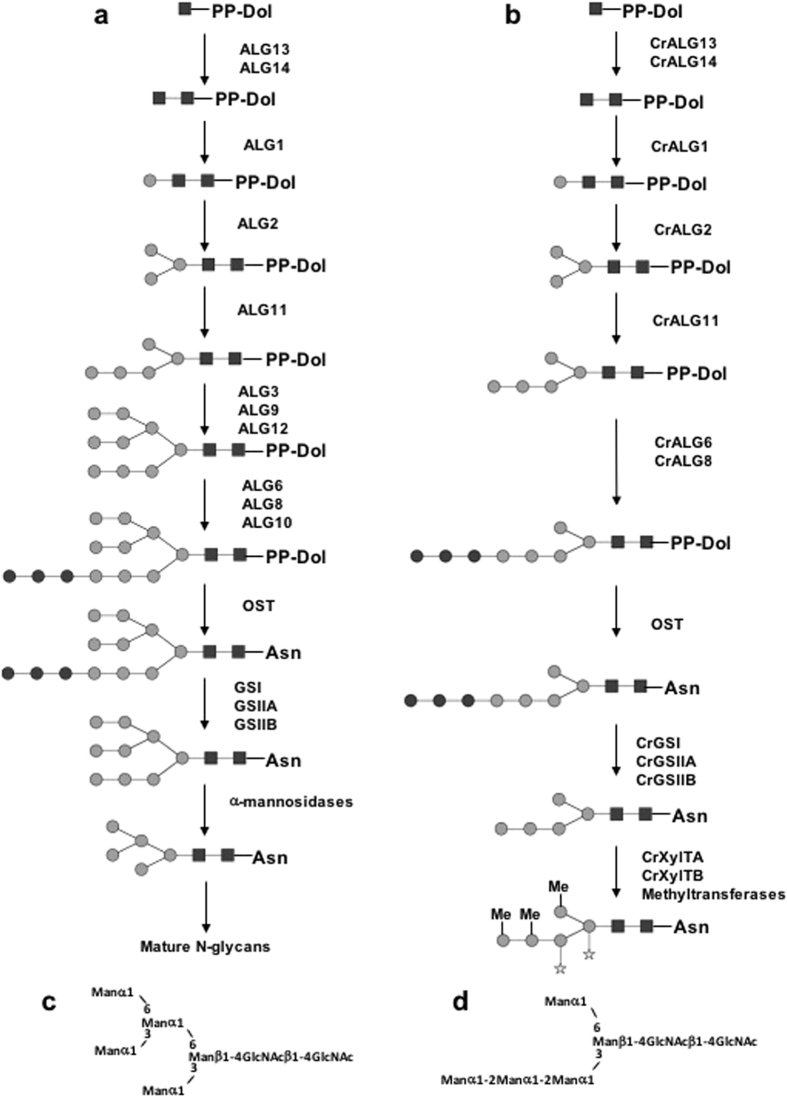
*N*-glycan biosynthesis in land plants and mammals (**a**) and proposed pathway in *C. reinhardtii* (**b**). Asn: asparagine residue of the *N*-glycosylation site (Asn-X-S/T/C). PP-Dol: dolichol pyrophosphate; Black square: GlcNAc; grey circle: Man; Star: xylose and Me: methyl substituent. Detailed structure of Man_5_GlcNAc_2_ in land plants and mammals (**c**) and in *C. reinhardtii* (**d**).

The structure of *C. reinhardtii* Man_5_GlcNAc_2_ isomer was further investigated using electrospray ionization-multistage tandem mass spectrometry (ESI-MS^n^) (with n = 2, n = 3 and n = 4) taking advantage of specific ion transitions reported in ref. 26 (Table 1). Fragmentation patterns of the doubly charged [M + 2Na]^2+^ of permethylated Man_5_GlcNAc_2_-2AB (*m/z* 882) prepared from proteins of PtGnTI#1 transformed line and from bovine ribonuclease B were compared (Fig. 4). The MS^3^ “*m/z* 882 → *m/z* 1302 → product ions” analysis of PtGnTI#1 *N*-glycans revealed the presence of discriminant product ions *m/z* 880 and *m/z* 676 (Fig. 4a, middle panel) that are not detected for the permethylated Man_5_GlcNAc_2_-2AB released from bovine ribonuclease B (Fig. 4b, middle panel). Moreover, the MS^4^ “*m/z* 882 → *m/z* 1302 → *m/z* 880 → product ions’’ analysis showed that *m/z* 880, which arose from the neutral loss of two terminal mannose residues from *m/z* 1302, fragments into *m/z* 676 (Fig. 4a, lower panel). Such a fragmentation pattern does not correspond to that of a branched Man_5_GlcNAc_2_ isomer, but to the one of a linear Man_5_GlcNAc_2_ structure^26^ (Table 1). Similar fragmentation patterns were observed for permethylated Man_5_GlcNAc_2_-2AB prepared from cw92 cells and AtGnTI cells (Fig. S4). In contrast, the fragmentation pattern deduced from the MS^4^ analysis of the *m/z* 882 for Man_5_GlcNAc_2_-2AB isolated from bovine ribonuclease B revealed the specific fragmentations *m/z* 882 → *m/z* 1302 → *m/z* 866 → *m/z* 648, as it is expected for the branched isomer of Man_5_GlcNAc_2_-2AB (Fig. 4b)^26^ (Table 1). In conclusion, the comparison of ESI-MS^n^ fragmentation patterns suggested that *C. reinhardtii* proteins carry linear Man_5_GlcNAc_2_ (Fig. 5d) instead of the conventional branched isomer (Fig. 5c).

**Table 1:**
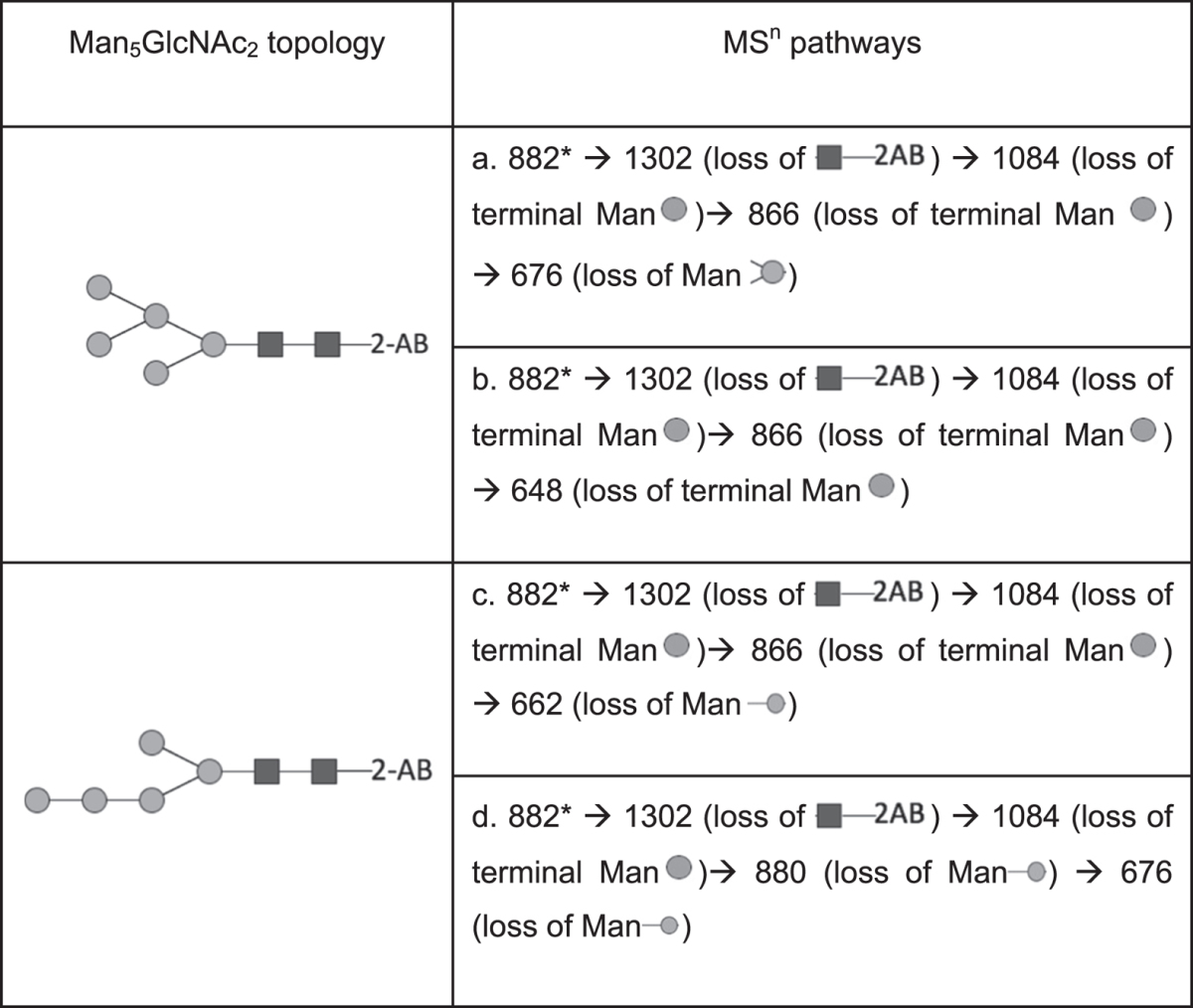
Transition fragmentation ions observed in ESI-MS^n^ for the branched and linear Man_5_GlcNAc_2_-2AB according to ref. 26. *The selected ion at *m/z* 882 corresponding to [M + 2Na]^2+^ precursor ion.

According to the *N*-glycan biosynthesis pathway described in land plants and mammals, a linear Man_5_GlcNAc_2_ is meant to exhibit terminal α(1,2/6)-Man residues instead of α(1,3/6)-Man. For further confirmation of the *C. reinhardtii* Man_5_GlcNAc_2_ topology, the *N*-glycan mixture was submitted to exoglycosidase degradations using either Jack bean mannosidase, a non-specific α-mannosidase and *Aspergillus saitoi* mannosidase, an exoglycosidase specific for α(1,2)-mannose residues^27^. Man_5_GlcNAc_2_-2AB from *C. reinhardtii* was efficiently converted into ManGlcNAc_2_-2AB by Jack bean mannosidase after removal of the four α-mannose units. In contrast, Man_3_GlcNAc_2_-2AB was the major end product after treatment with *Aspergillus saitoi* α(1,2)-mannosidase that results from the removal of two terminal α(1,2)-mannose residues from the linear trimannoside arm (Fig. S5). Taken together, both mass spectrometry and enzyme sequencing analyses demonstrated that *C. reinhardtii* glycoproteins carry *a* non-canonical linear Man_5_GlcNAc_2_, as depicted in Fig. 5d.

**Figure 6:**
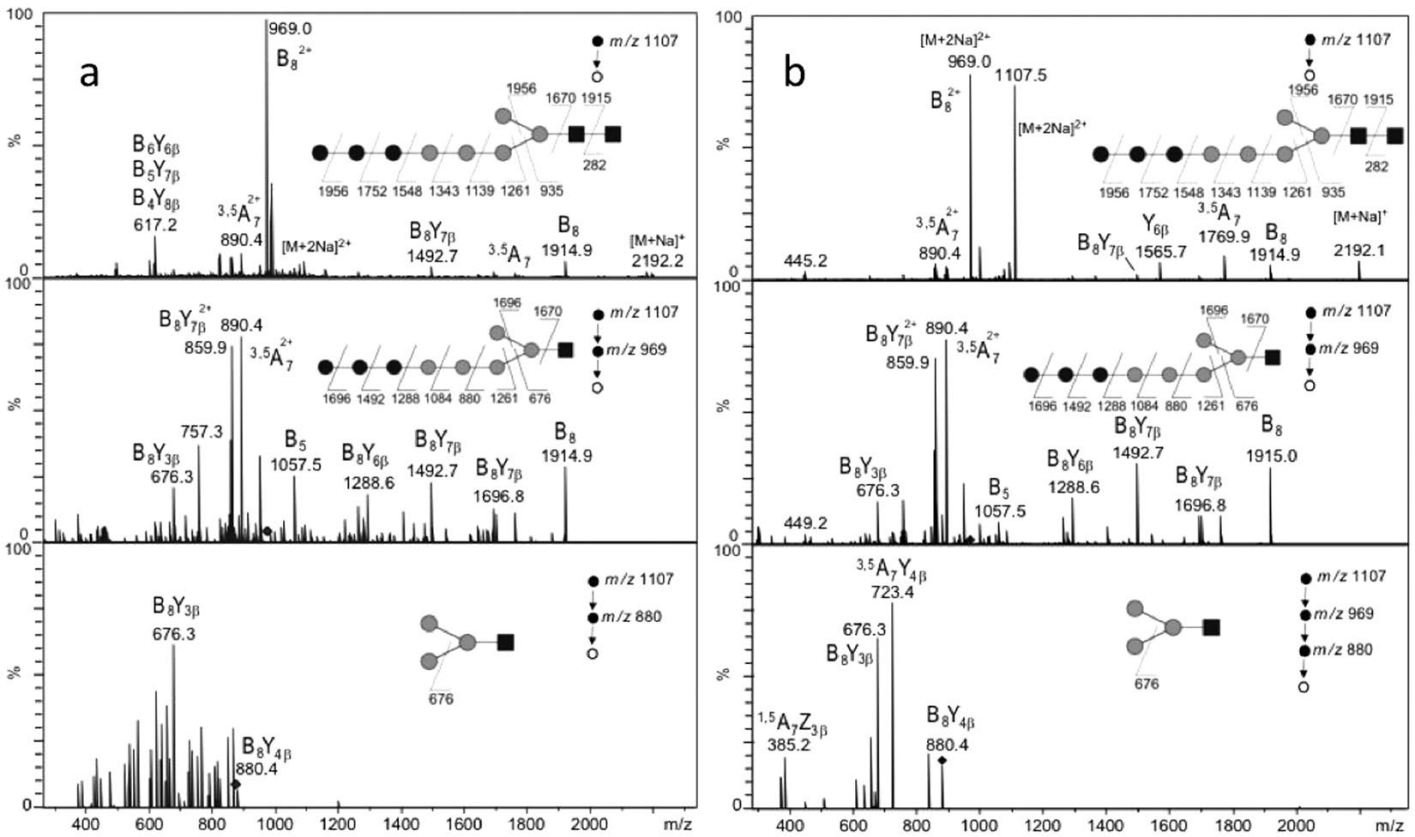
ESI-MS^n^ spectra with n = 2 (upper panel), n = 3 (middle panel + lower panel (a)) and n = 4 (lower panel (b)) of permethylated LLO derivative (*m/z* 1107 corresponding to [M + 2Na]^2+^ precursor ion) from lipid-linked oligosaccharides of cw92 *C. reinhardtii* cells (**a**) and the YG170 yeast mutant cells (**b**). On each panel, the precursor ion selected for the fragmentation analysis is shown with a diamond and its fragmentation pattern is proposed. Black square: GlcNAc; grey circle: Man; black circle: Glc.

The occurrence of a linear Man_5_GlcNAc_2_ oligosaccharide onto *C. reinhardtii* gycoproteins may result either from the trimming of Man_8-9_GlcNAc_2_ in the Golgi apparatus by the action of α-mannosidases or from a truncated biosynthesis of the LLO in the ER (Fig. 5). The latter hypothesis was investigated by isolation and analysis of *C. reinhardtii* LLO. LLO were isolated from cw92 microsomal preparations of *C. reinhardtii* using a methanol/chloroform extraction procedure according to a protocol adapted from^27, 28^. The oligosaccharide was hydrolyzed from the PP-dolichol anchor by mild acidic cleavage and then permethylated as previously reported^27, 29^. A predominant ion at *m/z* 2192.7 was observed using MALDI-TOF-MS and could be assigned to a permethylated oligosaccharide containing 8 hexoses and 2 HexNAc residues (Hex_8_HexNAc_2_) (Fig. S6). A minor ion at *m/z* 1987.6 could be assigned to Hex_7_HexNAc_2_ (Fig. S6). The sequence of this oligosaccharide was analyzed by ESI-MS^n^ and compared to the one isolated from LLO of the YG170 (*alg3*) yeast mutant (Fig. 6). Indeed, this strain lacks α1,3-mannosyltransferase ALG3 activity and accumulates Glc_3_Man_5_GlcNAc_2_ LLO in the ER^30, 31^. Both ESI-MS^n^ data exhibited similar fragmentation patterns that are consistent with a linear arrangement in the oligosaccharide. Taken together, these analyses demonstrated that *C. reinhardtii* accumulates a predominant linear truncated Glc_3_Man_5_GlcNAc_2_ LLO in the ER (Fig. 5b).

## Discussion

In previous work, we characterized *N-*glycan structures linked to endogenous proteins in *C. reinhardtii* and showed they were mostly oligomannosides and for 30% novel mature structures containing xylose residues and methylated mannoses on Man_4-5_GlcNAc_2_^15^. These structures resulted from a Golgi GnTI-independent processing of oligomannosides as the bioinformatics analysis of the genome has revealed that it lacks GnTI. Furthermore, no *N-*glycan harboring terminal GlcNAc residues has been identified in the whole glycan population^11, 15^. This situation contrasts with most eukaryotic organisms in which the GnTI is a key Golgi enzyme in the *N*-glycosylation pathway opening the door to the biosynthesis of structurally diverse mature *N-*glycans^12^. In this canonical pathway, the addition of a first GlcNAc by GnTI is required for the sequential activity of a large repertoire of other specific transferases giving rise to complex *N*-glycans involved in numerous biological processes, such as intracellular communication and signaling^13, 14^. Inactivation of GnTI in those organisms induces strong developmental phenotypes. For instance, GnTI-null embryos of mouse die at about 10 days after fertilization indicating that mature *N*-glycans are required for morphogenesis in mammals^32, 33^. Also, inactivation of GnTI reduces the viability in worm and fly^34, 35^. In plants, GnT I mutants exhibit a stress phenotype, thus suggesting a role for mature *N*-glycans in specific physiological processes^36, 37^. In rice, *gntI* mutants, impaired in the *N*-glycans maturation showed severe developmental defects, resulting in early lethality, associated to reduced sensitivity to cytokinin^36^.

*GnTI* genes are predicted in different microalgae genomes. In the diatom *P. tricornutum*, PtGnTI was demonstrated to encode for an active glycosyltransferase^17^. Therefore, the capacity of *C. reinhardtii* to express GnTI from Arabidopsis and from *P. tricornutum* was analyzed to investigate whether *C. reinhardtii N*-glycan biosynthesis can be complemented with this key transferase and shift into a GnTI – dependent pathway. Moreover, in a biotech context, the production, in microalgae, of recombinant glycoproteins for therapeutic applications will require the engineering of their endogenous *N*-glycosylation pathway for the production of biopharmaceuticals exhibiting human-compatible *N*-glycans. Therefore, implementation of a GnTI - dependent pathway is a prerequisite for any production of glycosylated biopharmaceuticals in *C. reinhardtii*.

Transgenic lines expressing the Arabidopsis or diatom GnTI were obtained and GnTI protein was immunodetected as expected in the microsomal fraction. However, further experiments are required to confirm the GnTI localization within the Golgi apparatus. Mass spectrometry analyses of *N*-glycan profiles from proteins secreted in transgenic lines did not show any modification of the *N-*glycan population by comparison with the cw92 cells. Among the different scenarios that may explain this result, the absence of UDP-GlcNAc nucleotide sugar in the Golgi apparatus was first considered. The transport of the cytosolic nucleotide sugars across the Golgi membrane is performed by Nucleotide Sugar Transporters (NSTs). Searching for NST orthologues in the *C. reinhardtii* genome allowed the identification of 23 candidate genes of which the deduced amino-acid sequences harbor the characteristic Triose Phosphate Translocator (TPT) domain (Pfam 03151) present in NSTs (Mathieu-Rivet *et al*., in press). However, as most of the characterized NSTs has been shown to transport at least two distinct substrates, determination of their specificity for nucleotide sugars based on sequence homologies with other Golgi NSTs remains difficult without additional biochemical evidence^38^. Therefore, further experimental work is needed to determine whether a specific Golgi UDP-GlcNAc transporter exists in *C. reinhardtii*. In relation to this, we cannot ruled out that the cytosolic abundance of UDP-GlcNAc may not be sufficient to supply the Golgi apparatus which would consequently limit GnTI in *C. reinhardtii*.

Inability of GnTI to affect the endogenous *N*-glycan population in *C. reinhardtii* transformed lines may also result from the absence of the appropriate glycan substrate. Man_5_GlcNAc_2_ oligomannoside was previously identified in *C. reinhardtii* ^15^ and considered as being the branched isomer substrate for GnTI by analogy with mammals and plants *N*-glycan pathways. Its structure was reinvestigated by mass spectrometry and enzyme sequencing and compared to a branched Man_5_GlcNAc_2_ standard from mammals^26^. Ion mobilities determined by IMS-MS and fragmentation patterns resulting from ESI-MS^n^ clearly show that *C. reinhardtii* and mammalian oligomannosides differ in their shape and sequence. Moreover, the fragmentation pattern determined by MS^n^ and enzyme digestion with an α(1,2)-mannosidase are consistent with the presence of a linear Man_5_GlcNAc_2_ on *C. reinhardtii* proteins as depicted in Fig. 5d. This oligosaccharide may result from a truncated LLO biosynthesis occurring in the ER (Fig. 5). Indeed, ALG3, ALG9 and ALG12 candidates are not predicted in the *C. reinhardtii* genome^15, 39, 40^. These ER enzymes are involved in the completion of the biosynthesis of the LLO precursor Man_9_GlcNAc_2_-PP-Dol prior to its glucosylation (Fig. 5a). As a consequence, the absence of ALG3, ALG9 and ALG12 activities results in the secretion, from ER, of proteins carrying a linear Man_5_GlcNAc_2_ instead of Man_8-9_GlcNAc_2_ (Fig. 5b). In contrast, in land plants and mammals, branched Man_5_GlcNAc_2_ is obtained by the trimming of mannose residues of Man_8-9_GlcNAc_2_ oligomannosides by Golgi α-mannosidases (Fig. 5a). However, linear Man_5_GlcNAc_2_ oligomannoside could also result from the trimming of Man_8-9_GlcNAc_2_ by Golgi α-mannosidases predicted in *C. reinhardtii* genome^11, 15^ but having different glycan specificities as compared to those of homologous enzymes involved in mammalian and plant *N*-glycan pathways. To discriminate between the two possibilities, *C. reinhardtii* LLO was isolated, characterized by mass spectrometry analysis and compared with the one extracted from the ALG3 deficient yeast mutant^31^. These analyses demonstrated that this microalga accumulates a linear Glc_3_Man_5_GlcNAc_2_ (Fig. 6 and Fig. S6). Such a truncated ER pathway has been already characterized in some unicellular organisms such as the coccidian parasites Toxoplasma and Cryptosporidium^41–45^. In conclusion, data obtained in this study revealed a truncated ER *N*-glycan pathway in *C. reinhardtii* and required the reevaluation of the previously published *N*-glycan pathway^15^. In this reassessed *N*-glycan processing, Man_5_GlcNAc_2_ results from the deglucosylation of Glc_3_Man_5_GlcNAc_2_ precursor in the ER and its methylation and xylosylation in the Golgi apparatus (Fig. 5). Location of xylose residues on Man_5_GlcNAc_2_ was reinvestigated by ESI-MS^n^ (Fig. S7). This analysis confirmed the presence of the first xylose residue onto the β-Man^15^. In addition, the second xylose could be positioned either on the α(1, 3)-linked Man (I) or the first α(1, 2)-linked Man (II). MS^4^ experiments are more consistent with the structure I depicted in Fig. 5.

Surprisingly, cells expressing *GnTI* exhibited an altered phenotype although no modification of the *N-*glycans has been observed. The presence of large vacuoles as well as the increase of ROS production observed in transformed cell lines suggested that transformation with *GnTI* induced the activation of stress responses such as autophagy and oxidative stress^46, 47^. Previously, Pérez-Martin and collaborators showed that ER stress caused by tunicamycin or DTT triggers autophagy^48^. It can be hypothesized that AtGnTI or PtGnTI may localize in the ER of *C. reinhardtii*. Indeed, a previous study published by Schoberer and coworkers in 2009 demonstrated that overexpressed GnTI in tobacco recruit the ER protein Sar1p resulting in a GnTI-Sar1p association in the ER membranes^49^.

In addition, chlamydomonas cells expressing At*GnTI* or Pt*GnTI* accumulate starch. In BY2 cells and *chlamydomonas noctigama*, Hummel and collaborators^50^ reported in 2010 that the disassembly of the Golgi apparatus using the secretion inhibitor Brefeldin A (BFA) caused the accumulation of plastid starch. This starch increase was also observed when the CopII-mediated ER to Golgi transport was inhibited in tobacco plants expressing a dominant negative version of the small GTPase Sar1p^51^. Moreover, it was proposed that disruption of the Golgi apparatus resulting in the loss of secretory activity may cause the redirection of free carbohydrates to the plastids where they would be converted into starch^51^.

Therefore, we postulate here that the heterologous expression of *GnTI* may affect Golgi organization or the endosomal system, which would consequently disturb the biosynthesis of glycans or glycoconjugates and the cell biology of *C. reinhardtii*. Additional work and phenotypic studies of transformants expressing a non-functional GnTI would allow the confirmation of this hypothesis.

## Methods

### Strains and growth conditions

CC-503 cw92 strain, later called cw92 cells, was obtained from the Chlamydomonas Culture Collection at Duke University (Durham, NC, USA) and grown in batch cultures at 25 °C, illuminated with 150 μmol m^−2^ s^−1^ using TAP medium redirection of free carbohydrates to the plastids where they would be converted into starch^52^.

### Vector construction and transformation

The sequences encoding for At*GnTI* (At4g38240) and Pt*GnTI* (gi: 307604450) fused to the V5 epitope^17^ were codon optimized and synthesized by Sloning Biotechnology. Optimized sequences were cloned as a XbaI/NdeI fragment in the pSL18 vector^53^. pSL18 (empty vector), pSL18-At*GnT*I or pSL18-Pt*GnT*I construct was introduced using the glass beads method^54^. To detect the transgene in genomic DNA, screening was performed by PCR using V5-reverse GGAGTCCAGGCCCAGCAGG and with At*GnTI*-forward GCCCTAAGTGGCCAAGGC or Pt*GnT1*-forward CCAGTCCAAGTGGCCGGGC as primers.

### RT-PCR analysis

Total RNA were isolated from fresh cell pellets (1.5 × 10^6^ cells), using TRIzol (Invitrogen). gDNA contamination was removed by a Turbo DNase treatment (Thermo Fisher Scientific). Reverse transcription was performed on 2 µg of RNA using the High-Capacity cDNA Reverse Transcription Kit (Thermo Fisher Scientific). The transcription level of At*GnTI* and Pt*GnTI* transgenes was analyzed by PCR using the V5-reverse primer, with either At*GnTI*-forward or Pt*GnT1*-forward primers. Each PCR contained 2 µL of diluted cDNA (1/10), 0.8 mM of each primer, 0.25 mM dNTPs and 0.6 U GoTaq polymerase (Promega) and was performed in the reaction buffer provided by the manufacturer, according the following program: 95 °C for 5 min, 35 cycles of 95 °C for 30 s, 62 °C for 30 s, 72 °C for 30 s and a final step of 72 °C for 5 min. The expression of the actin gene was monitored as a control using CrActin-forward CGCTGGAGAAGACCTACGAG and CrActin-reverse GGAGTTGAAGGTGGTGTCGT as primers.

### Microsomal preparations and Western-blot analysis

1.4 × 10^7^ of *C. reinhardtii* cells were collected (2,500 g for 5 min) and washed with a 20 mM potassium phosphate buffer at pH 7.4. All the following preparation steps were carried out at 4 °C. The cell pellet was broken with 2 mL of protease inhibitor cocktail 25X (Roche) dissolved in 10 mM potassium phosphate buffer (pH 7.4) using the FastPrep-24™ 5G^15^. Samples were then spun (300 g for 3 min) in order to remove intact cells and debris. The supernatant was collected and centrifuged (20,000 g for 30 min) in order to eliminate pigments and chloroplasts. Finally, the supernatant was ultracentrifuged (100,000 g for 1 h) to pellet the microsomal fraction. Immunodetection using anti-V5 antibodies has been performed as described in ref. 17.

### *N*-glycans preparation and derivatization

Total proteins were extracted and deglycosylated using Peptide *N-*glycosidase F (PNGase F) as described in ref. 15. The released *N*-glycans were then labeled with 2-aminobenzamide (2AB) according to ref. 55. Excess of reagent was removed using a cartridge D1 from Ludger. Freeze dried samples were resuspended in 10 µL of water. Jack bean mannosidase (Sigma M7944) or *Aspergillus saitoi* α(1,2)-mannosidase (ProZyme) enzymatic treatments were performed on 4.5 µL of 2AB-labeled *N*-glycans according to the manufacturer’s instructions.

*Permethylation -* The 2AB-labeled *N-*glycans were permethylated^29^ and cleaned-up according to ref. 56.

### LC-ESI-MS

The analyses were performed using the nano-LC1200 system coupled to a QTOF 6550 mass spectrometer equipped with a nanospray source and a LC-Chip Cube interface (Agilent Technologies, les Ulis, France). Briefly, 2AB-labeled *N-*glycans were enriched and desalted on a 500 nL PGC trap column and separated on a PGC (3-μm particle size) column (150 mm long × 75 μm inner diameter, Agilent Technologies). A 30-min linear gradient (5–75% acetonitrile in 0.1% formic acid) at a flow rate of 400 nL min^−1^ was used. Separated *N*-glycans were analyzed with the QTOF analyser. Full autoMS scans from 290 to 1,700 m/z and autoMS/MS from 59 to 1,700 *m/z* were recorded. In every cycle, a maximum of 5 precursors sorted by charge state (1+ and 2+ preferred) were isolated and fragmented in the collision cell with fixed collision cell energy at 15 eV. Scan speed raise based on precursor abundance (target 5,000 counts/spectrum) and precursors sorted only by abundance. Active exclusion of precursors was enabled and the threshold for precursor selection was set to 1,000 counts. The ESI acquisition parameters in positive mode were: capillary voltage; 1.9 kV, drying gas temperature; 250 °C, gas flow (air); 11 L min^−1^; fragmentor voltage, 360 V; Skimmer1 voltage, 65 V and OctopoleRFPeak voltage, 750 V.

### IM-MS analyses

The ESI-IM-MS experiments were performed using a Waters SYNAPT G2 hybrid quadrupole/HDMS instrument equipped with an ESI LockSpray™source, the MassLynx 4.1 and the DriftScope 2.2 softwares (Waters, Manchester, UK). The SYNAPT HDMS system was calibrated using sodium formate cluster ions (2 mg mL^−1^) and operated in ‘V’ resolution mode (resolution 20,000 FWHM). The ESI parameters were in positive ion mode: capillary voltage, 3 kV; sample cone voltage, 70 V; source temperature, 90 °C; desolvation temperature, 250 °C; desolvation gas flow (N_2_), 700 L h^−1^. The data were acquired using a 50–2000 *m/z* range with 1 s scan time and 0.02 s interscan delay. Sample solutions were infused into the source at a flow rate of 400 µL h^−1^ with a syringe pump (Cole-Palmer, Vernon Hills, Illinois, USA). The IMS conditions were optimized as followed: gas flow (N_2_), 90 mL min^−1^; IMS pressure, 3.05 mbar; wave height voltage, 40 V and T-wave velocity, 550 m s^−1^. The ion mobility spectra were fitted using the Origin 9.0 software (OriginLab).

### ESI-MS^n^ analyses

Permethylated 2AB-derivatives of *N*-glycans were analyzed by ESI-MS^n^ (n = 1 to 4) using a Bruker HCT Ultra ETD II quadrupole ion trap (QIT) mass spectrometer equipped with the Esquire control 6.2 and Data Analysis 4.0 softwares (Bruker Daltonics, Bremen, Germany). The ESI parameters were as followed: capillary and end plate voltages respectively set at −3.5 kV and −3.0 kV in positive ion mode, skimmer and capillary exit voltages set at 40 V and 200, respectively, nebulizer gas (N_2_), pressure, drying gas (N_2_) flow rate and drying gas temperature were 10 psi, 7.0 L min^−1^ and 300 °C, respectively. Helium pressure in the ion trap was 1.2 × 10^−5^ mbar. The data were acquired using a 200–2200 *m/z* range, using a scan speed of 8,000 m/z per second. The number of ions entering the trap cell was automatically adjusted by controlling the accumulation time using the ion charge control (ICC) mode (target 200,000) with a maximum accumulation time of 50 ms. The injection low-mass cut-off (LMCO) value was *m/z* 100 for Man_5_GlcNAc_2_ derivatives and 140 for LLO precursor respectively. The values of spectra averages and rolling average were 6 and 2. ESI-MS^n^ experiments were carried out by collision-induced dissociation (CID) using helium as the collision gas, isolation width of 2 *m/z* unit for the precursor ions and for the intermediate ions using a resonant excitation frequency with an amplitude from 0.8 to 1.0 Vp–p. Sample solutions were infused into the source at a flow rate of 300 µL h^−1^ by means of a syringe pump (Cole-Palmer, Vernon Hills, IL, USA).

### Electron microscopy

High pressure freezing was performed with the freezer HPM100 Leica-microsystems. Prior to freezing, 72 h old *C. reinhardtii* cells were treated at room temperature 1 hour with 100 mM mannitol as cryoprotectant diluted in fresh culture medium. Pre-treated *C. reinhardtii* cells were transferred into an aluminium cryocapsule covered by soy lecithin dissolved at 100 mM in chloroform. Excess medium was absorbed by filter paper. After fixation on the loading device, samples were frozen according to a maximum cooling rate of 20,000 °C s^−1^ and a pressure of 2,100 bars. Samples were transferred to a freeze substitution automate (AFS2, Leica) pre-cooled to −110 °C. Samples were substituted in anhydrous acetone with 0.5% uranyl acetate and 0.5% osmium tetroxide at −90 °C for 72 h. The temperature was gradually raised to −60 °C using a gradient of +2 °C h^−1^ and stabilized during 12 h, then gradually raised to −30 °C by using the same gradient during 12 h and gradually raised again to +4 °C. Then, samples were rinsed twice at room temperature with anhydrous acetone. Infiltration was then processed in acetone-Spurr’s resin (Spurr 25%, 8 h at +4 °C; Spurr 50%, 16 h at +4 °C; Spurr 75%, 8 h at room temperature; Spurr 100%, 16 h at room temperature; Spurr 100%, 24 h at room temperature). The Spurr’s resin was finally polymerised at +50 °C during 24 h. Ultrathin sections (70 nm; ultracut UC6, Leica) were collected onto carbon-formvar-coated nickel grids. A classical staining using 0.5% uranyl acetate and 0.5% lead citrate was done before sections were observed in a Philips, FEI Tecnai 12 Biotwin transmission electron microscope operating at 80 kV, with ES500W Erlangshen CCD camera (Gatan).

### Size measurements

Cells (1.5 × 10^7^) were collected (4,500 g for 5 min). The cell pellet was incubated in TAP medium containing 4% of paraformaldehyde (PAF) (v/v) during 30 min, under shaking, at room temperature. Then, cells were washed four times and finally resuspended in 200 µL of TAP. 20 µL of fixed cells were spotted between slide and coverslip (Superfrost™Plus, Thermo Scientific) and were observed with a Leica DMI-6000B inverted microscope at magnification 400. Cell size was determined using fixed cells by measuring the cell longitudinal diameter with Image J software. The measurements were done in triplicates on a minimum of 200 cells (n > 200).

### Reactive oxygen species measurement

Reactive oxygen species (ROS) production was evaluated by electron paramagnetic resonance (EPR) spectroscopy. Cells were incubated at room temperature in the dark for 60 min in Krebs-HEPES buffer containing 5 µM diethyl dithiocarbamate, 25 µM deferoxamine, and the spin probe 1-hydroxy-3-methoxycarbonyl-2,2,5,5-tetramethyl pyrrolidine hydrochloride (CMH; 500 µM; Noxygen, Elzach, Germany). Spectra of the oxidized product of CMH (CM.) were recorded from frozen samples with a X-band spectrometer (MS-400; Magnetech, Berlin, Germany) with the following acquisition parameters: microwave power, 1 mW; modulation amplitude, 5 G; sweep time, 60 s; and 1 scans. After correction of the baseline, the total amplitude of the signal was measured and expressed in arbitrary units produced per 12 × 10^4^ cells for 60 min. After EPR analyses, total chlorophyll was quantified for each sample according to ref. 57. The quantity of total chlorophyll was used to normalize the signal intensity evaluated by EPR for each sample. The results were statistically analyzed using GraphPad Prism® software. The ROS level in each transformed cell line was normalized against ROS level measured in cw92 cells. To determine the significant level, a statistical test was performed between the GnTI transformed cell lines and the lines transformed with the empty vector using Ordinary One-Way ANOVA with n = 3 and p-value fixed at 0.05.

### LLO preparation

The LLO extraction was performed on microsomal fractions from *C. reinhardtii* according to a protocol adapted from^27, 28^. Released oligosaccharides were then lyophilized and resuspended in 500 µL of water and purified on a carbograph column (Hypersep Hypercarb, Thermo Scientific) using the manufacturer’s instructions.

## Electronic supplementary material

Supplemental Figures
