# Supplementary material for: Heterologous expression of the N-acetylglucosaminyltransferase I dictates a reinvestigation of the N-glycosylation pathway in Chlamydomonas reinhardtii
Source: Sci Rep. 2017 Aug 31;7:10156. doi: 10.1038/s41598-017-10698-z (PMC5578997; doi:10.1038/s41598-017-10698-z)
Supplement: Supplementary file 1 — Supplemental Figures [file 41598_2017_10698_MOESM1_ESM.pdf]

Heterologous expression of the *N*-acetylglucosaminyltransferase I dictates a reinvestigation of the *N*-glycosylation pathway in *Chlamydomonas reinhardtii*

Gaëtan Vanier*, Pierre-Louis Lucas*, Corinne Loutelier-Bourhis, Jessica Vanier, Carole Plasson, Marie-Laure Walet-Balieu, Philippe Chan Tchi-Song, Isabelle Remy-Jouet, Vincent Richard, Sophie Bernard, Azeddine Driouich, Carlos Afonso, Patrice Lerouge, Elodie Mathieu-Rivet and Muriel Bardor

* Equal contribution of the first two authors.

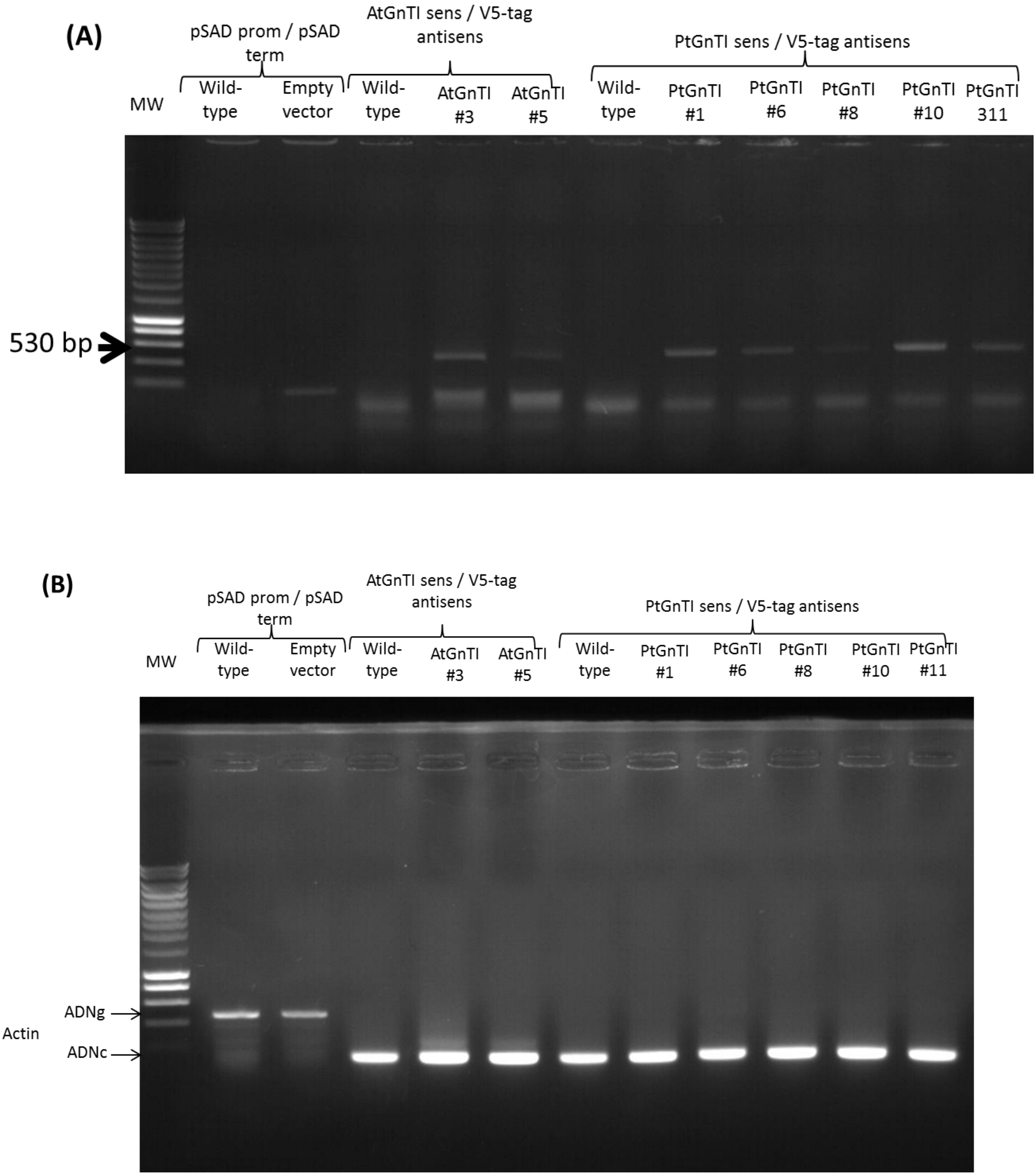

Figure S1 Panel (A) : RT-PCR analysis of AtGnTI (AtGnTI for/V5-tag rev) and PtGnTI (PtGnTI for/ V5-tag rev) transcription level in cw92 strain (WT), cells transformed with the empty vector (EV) and cells transformed with AtGnTI (lines At#3 and At#5) or PtGnTI (lines Pt#1, Pt#6, Pt#8, Pt#10 and Pt#11). Both oligonucleotide couples amplified approximatively a 530 bp fragment. As negative controls, the amplification of the empty cassette (using oligonucleotides pSAD prom / pSAD term specific of the promoter and terminator, respectively) was performed on cDNA from WT and EV (gel lines 1 and 2). The transcription level of transgenes was analyzed relatively to those of the actin gene as a reference: **Panel (B)**, gel lines 3 to 9). The amplification of actin on gDNA from WT and EV (gel lines 1 and 2) revealed a fragment of higher molecular mass, due to the presence of an intron. MW: molecular weight.

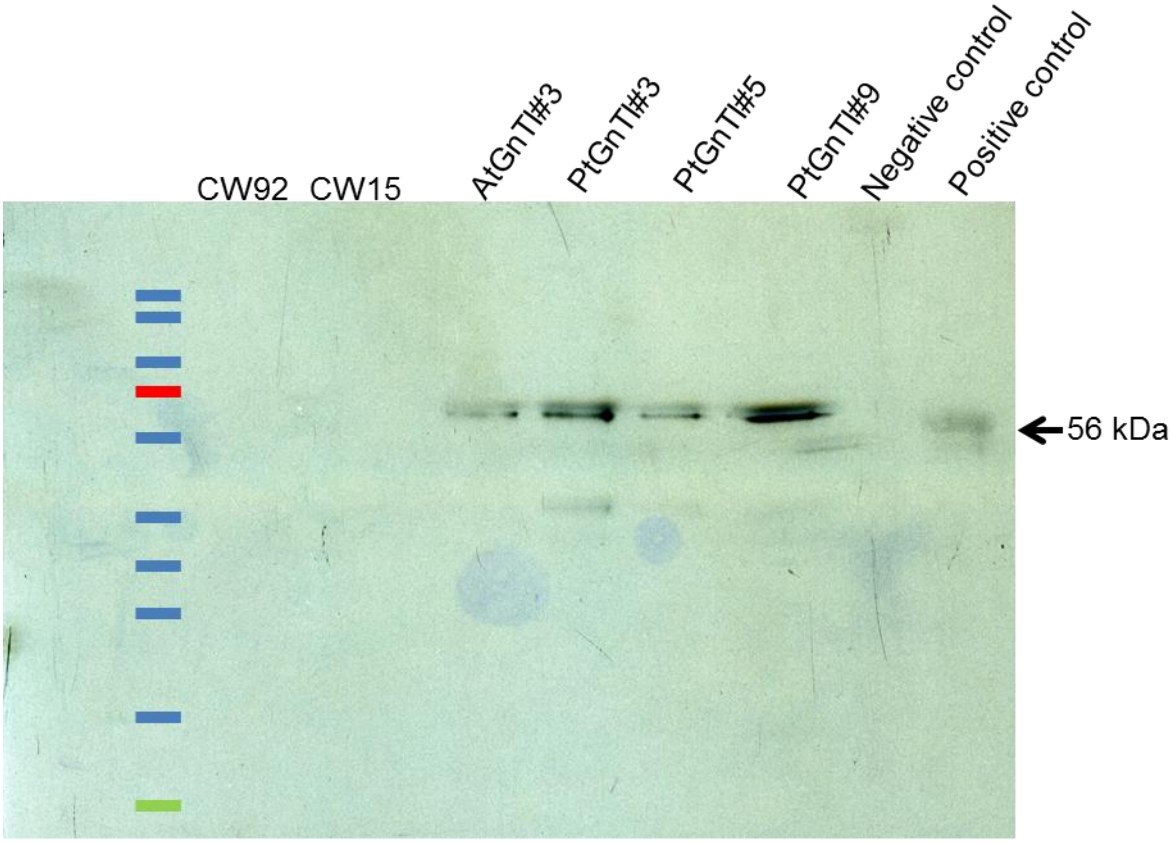

Figure S2: Immunodetection of recombinant GnTI in the microsomal fraction isolated from cw92 cells, cw15 cells and the different lines expressing the AtGnTI and PtGnTI respectively. The immunodetection was performed using an anti-V5 antibody as a primary antibody. A protein extract from CHO cells expressing *PtGnTI-V5* (+) was used as a positive control[19]. MW: molecular weight.

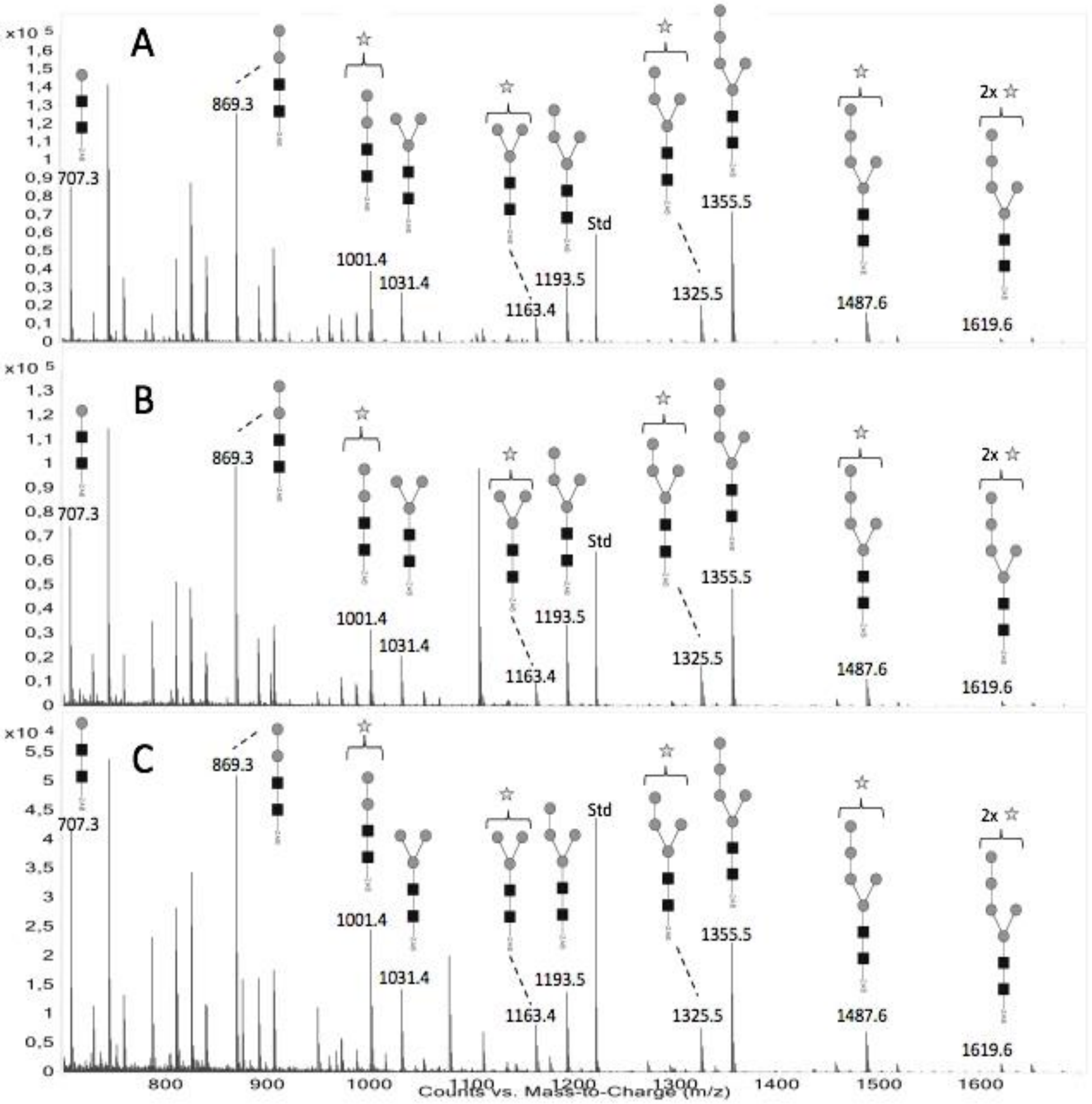

Figure S3. LC-ESI-MS profiles of 2AB derivatives of *N*-glycans isolated from *C. reinhardtii* cw92 cells (a) and transformed lines expressing AtGnTI#3 (b) or PtGnTI#10 lines (c). 2AB: 2-aminobenzamide derivative; Std: internal standard; Black square: GlcNAc; grey circle: Man; star: Xyl.

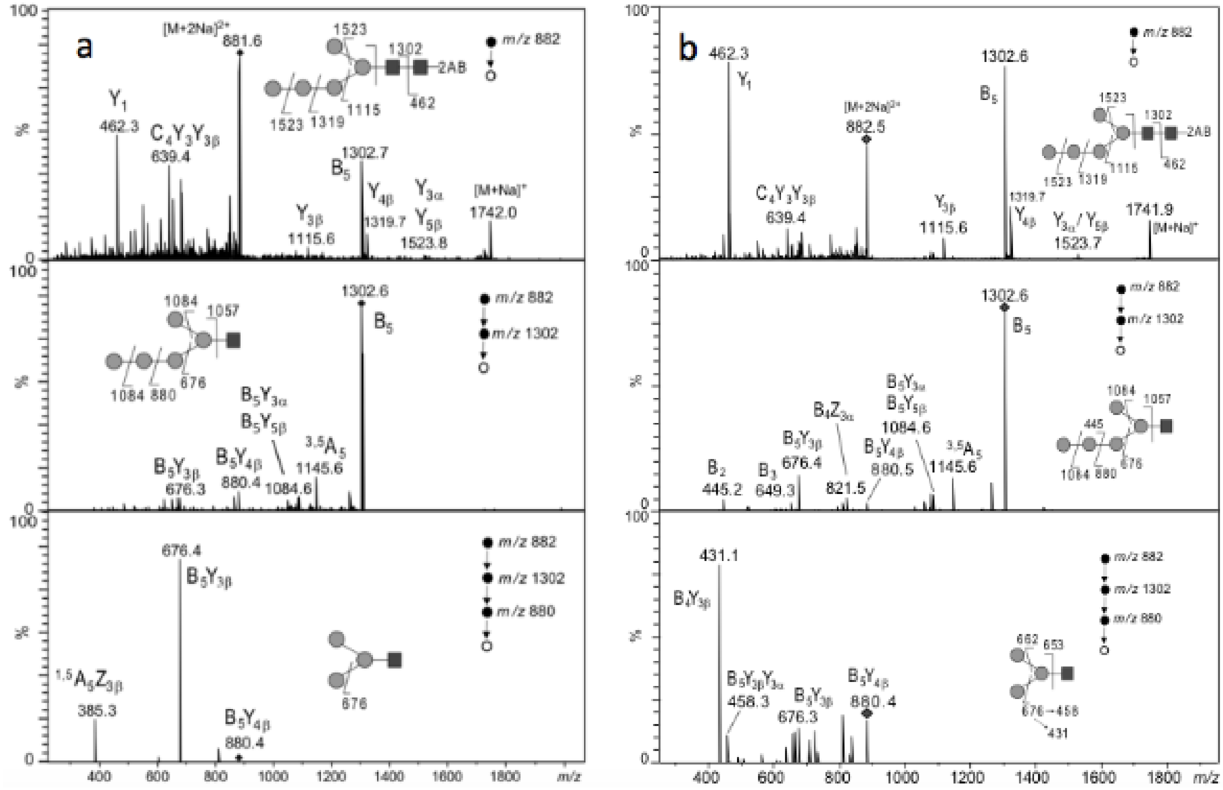

Figure S4. ESI-MS^n spectra with n = 2 (upper panel), n = 3 (middle panel) and n = 4 (lower panel) of permethylated $Man_5GlcNAc_2$-AB derivative (*m/z* 882 corresponding to $[M+2Na]^{2+}$ precursor ion) isolated from (a) cw92 *C. reinhardtii* proteins, (b) AtGnTI *C. reinhardtii* transformant proteins. On each panel, the precursor ion selected for the fragmentation analysis is shown with a diamond and its fragmentation pattern is proposed according to[28] for a linear $Man_5GlcNAc_2$ structure. 2AB: 2-aminobenzamide derivative; Black square: GlcNAc; grey circle: Man.

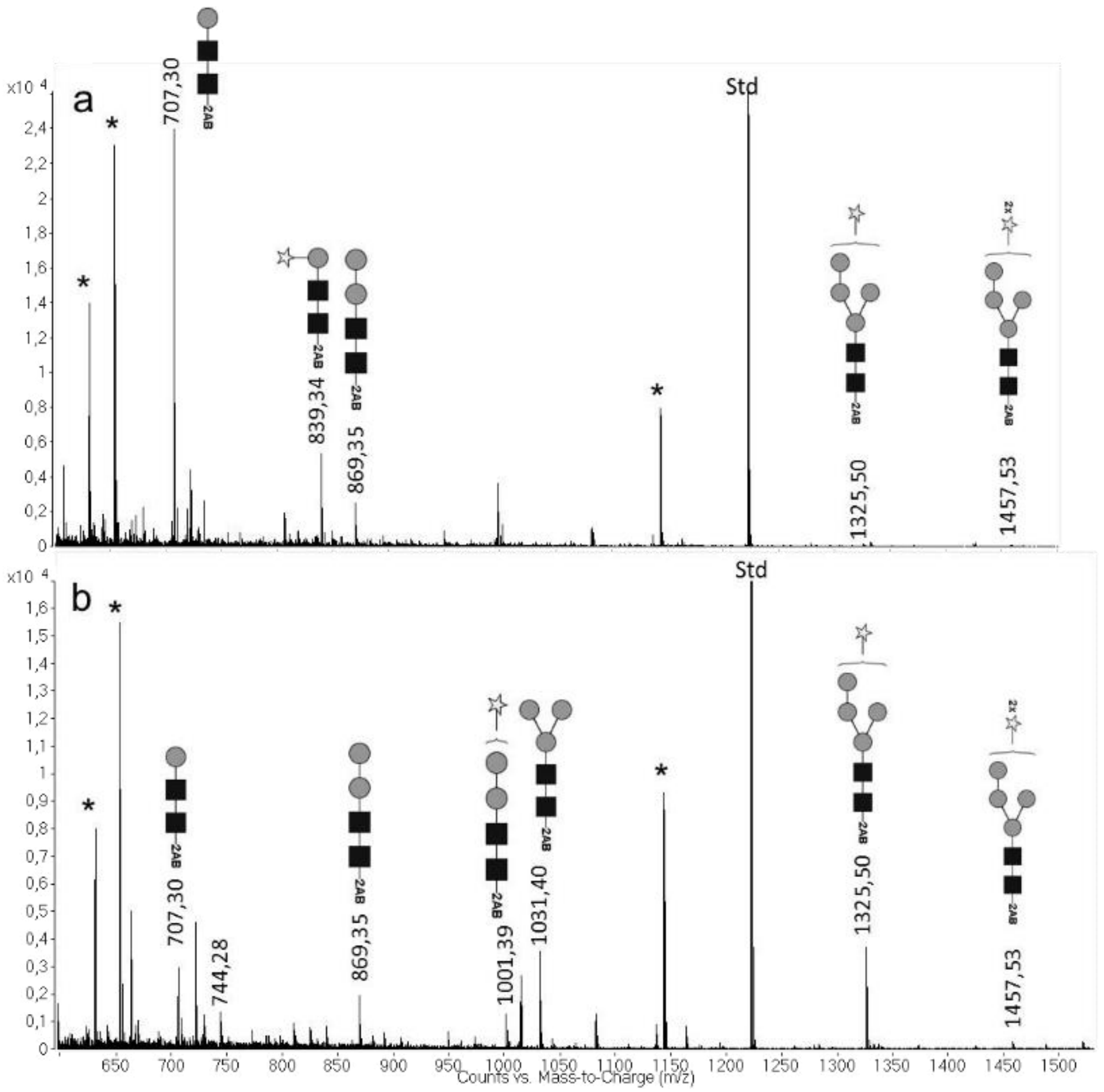

Figure S5. LC-ESI-MS profiles of 2AB derivatives of *N*-glycans isolated from *C. reinhardtii* cw92 cells after treatment with the Jack bean α-mannosidase (a) and the *Aspergillus saitoi* α(1,2)-mannosidase (b). *: contaminants. 2AB: 2-aminobenzamide derivative; Std: internal standard; Black square: GlcNAc; grey circle: Man; star: Xyl.

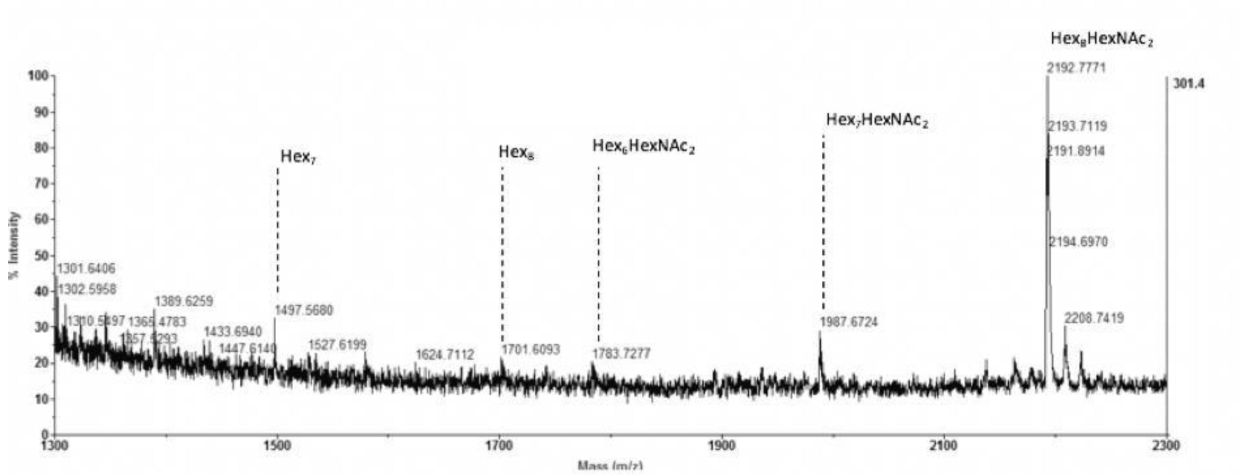

Figure S6. MALDI-TOF-MS profile of permethylated LLO isolated from *C. reinhardtii* cw92 cells.

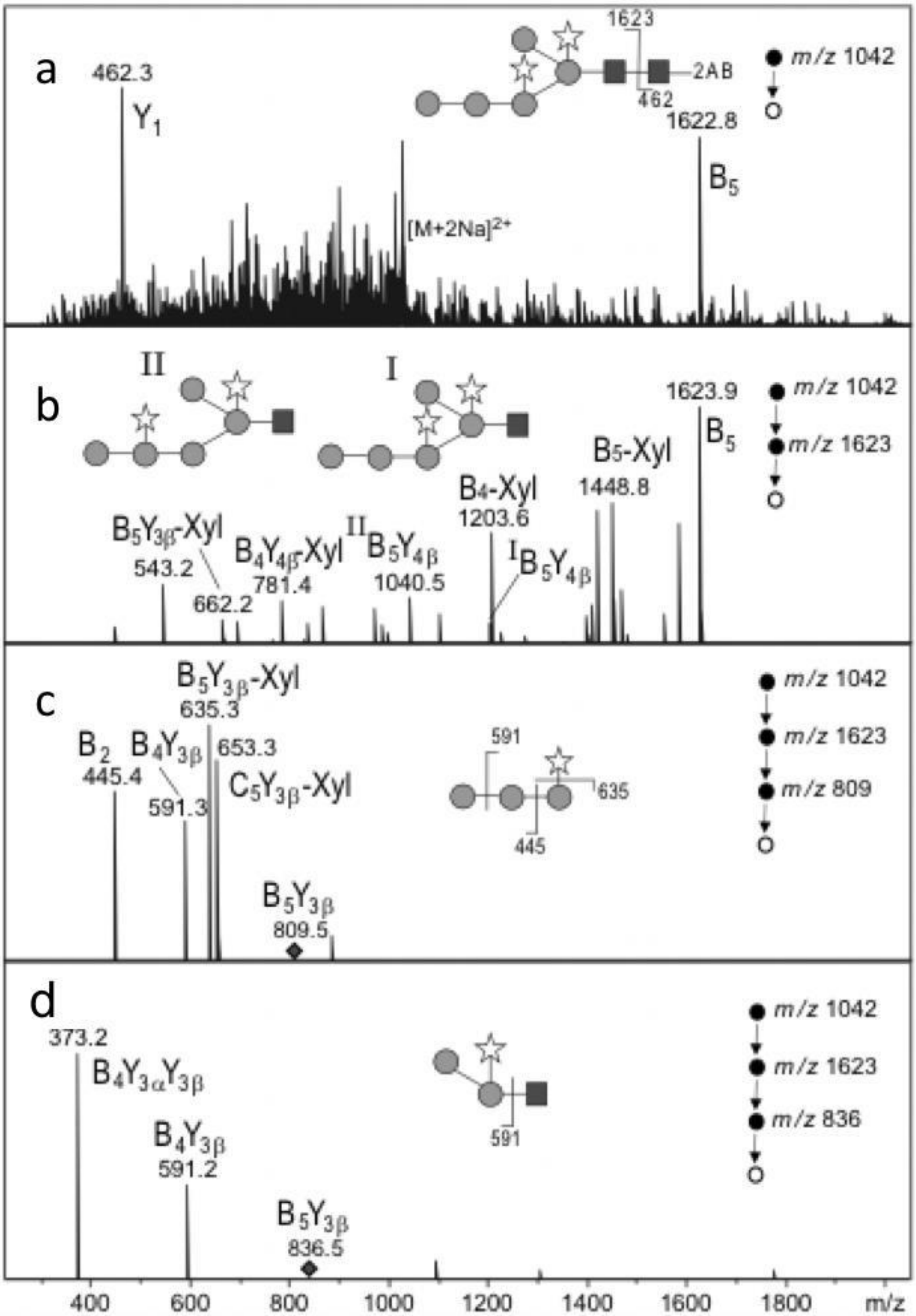

Figure S7. ESI-MS^n spectra with n = 2 (a), n = 3 (b) and n = 4 (c and d) of permethylated $Man_5GlcNAc_2Xyl_2$-2AB derivative (*m/z* 1042 corresponding to $[M+2Na]^{2+}$ precursor ion) isolated from cw92 cells. On each panel, the precursor ion selected for the fragmentation analysis is shown with a diamond and its fragmentation pattern is proposed. 2AB: 2-aminobenzamide derivative; Black square: GlcNAc; grey circle: Man; star: Xyl.
